# 2-[2-(4-Methyl­piperazin-1-yl)eth­yl]iso­indoline-1,3-dione

**DOI:** 10.1107/S1600536814002232

**Published:** 2014-02-12

**Authors:** Mi Zhou, Ying Shao, Yong-an Xia, Xiao-Long Liu, Xiao-Qiang Sun

**Affiliations:** a Key Laboratory of Fine Petrochemical Engineering, Changzhou University, Changzhou 213164, Jiangsu, People’s Republic of China

## Abstract

In the title compound, C_15_H_19_N_3_O_2_, the piperazine ring adopts a chair conformation, with its N—C bonds in pseudo-equatorial orientations. The dihedral angle between the C atoms of the piperazine ring and the phthalamide ring system (r.m.s. deviaiton = 0.008 Å) is 89.30 (8)°. In the crystal, mol­ecules are linked by C—H⋯O hydrogen bonds, generating a three-dimensional network and aromatic π–π inter­actions also occur [centroid–centroid distances = 3.556 (1)–3.716 (1) Å].

## Related literature   

For background to piperazine derivatives, see: Tian *et al.* (2011 ▶); Stauffer (2011 ▶). For the preparation, see: Ghosh *et al.* (2010 ▶). For a similar structure, see: Shao *et al.* (2012 ▶).
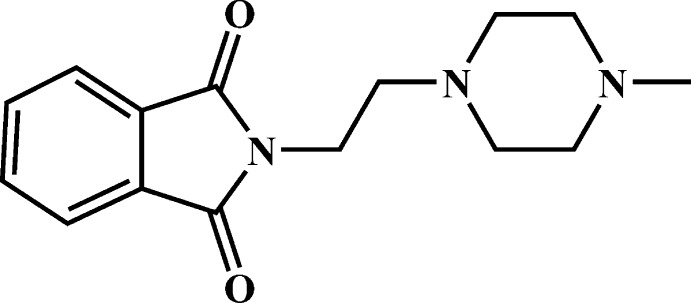


## Experimental   

### 

#### Crystal data  

C_15_H_19_N_3_O_2_
*M* *_r_* = 273.33
Triclinic, 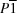

*a* = 6.9537 (12) Å
*b* = 8.4410 (15) Å
*c* = 12.563 (2) Å
α = 96.260 (4)°
β = 98.381 (4)°
γ = 92.647 (3)°
*V* = 723.7 (2) Å^3^
*Z* = 2
Mo *K*α radiation
μ = 0.09 mm^−1^
*T* = 296 K
0.30 × 0.28 × 0.25 mm

#### Data collection  

Bruker APEXII CCD diffractometer
Absorption correction: multi-scan (*SADABS*; Bruker, 2000 ▶) *T*_min_ = 0.975, *T*_max_ = 0.979
4194 measured reflections
2654 independent reflections
2206 reflections with *I* > 2σ(*I*)
*R*_int_ = 0.022

#### Refinement  

*R*[*F* ^2^ > 2σ(*F* ^2^)] = 0.047
*wR*(*F* ^2^) = 0.154
*S* = 1.01
2654 reflections
183 parameters
H-atom parameters constrained
Δρ_max_ = 0.17 e Å^−3^
Δρ_min_ = −0.18 e Å^−3^

### 

Data collection: *APEX2* (Bruker, 2000 ▶); cell refinement: *SAINT* (Bruker, 2000 ▶); data reduction: *SAINT*; program(s) used to solve structure: *SHELXS97* (Sheldrick, 2008 ▶); program(s) used to refine structure: *SHELXL97* (Sheldrick, 2008 ▶); molecular graphics: *SHELXTL* (Sheldrick, 2008 ▶); software used to prepare material for publication: *SHELXTL*.

## Supplementary Material

Crystal structure: contains datablock(s) I, New_Global_Publ_Block. DOI: 10.1107/S1600536814002232/hb7071sup1.cif

Structure factors: contains datablock(s) I. DOI: 10.1107/S1600536814002232/hb7071Isup2.hkl

Supporting information file. DOI: 10.1107/S1600536814002232/hb7071Isup3.cdx

Supporting information file. DOI: 10.1107/S1600536814002232/hb7071Isup4.cml

CCDC reference:

Additional supporting information:  crystallographic information; 3D view; checkCIF report

## Figures and Tables

**Table 1:** Hydrogen-bond geometry (Å, °)

*D*—H⋯*A*	*D*—H	H⋯*A*	*D*⋯*A*	*D*—H⋯*A*
C3—H3⋯O1^i^	0.93	2.57	3.406 (3)	149
C15—H15*B*⋯O2^ii^	0.96	2.53	3.343 (3)	142

Symmetry codes: (i) 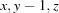; (ii) 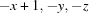.

**Fig. 1.**
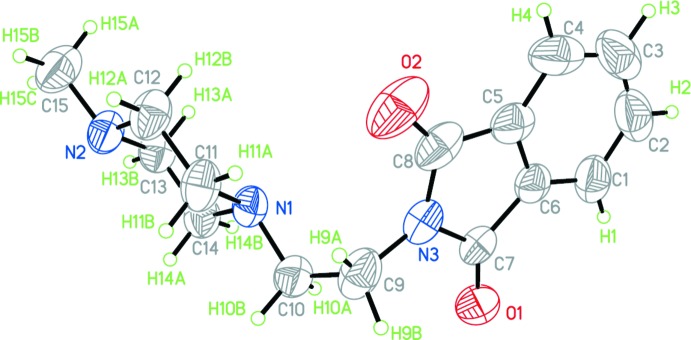
View of the title compound, showing 50% probability ellipsoids.

**Fig. 2.**
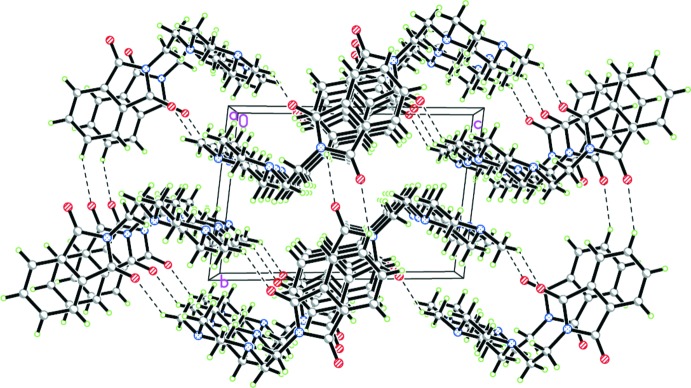
Perspective view of the title compound along a direction. Labels of atoms have been omitted for clarity.

## supplementary crystallographic information

### 1. Comment

Piperazine derivatives have received much attention for their pharmaceutical activities (Tian, *et al.*, 2011; Stauffer, 2011). The title compound was synthesized from *N*-(2-bromoethyl)phthalimide and *N*-methylpiperazine (Ghosh, *et al.*, 2010), as a drug intermediate. In the molecule, Fig. 1, the six-membered piperazine ring adopts the chair conformation. The phthalimide ring is coplanar [r.m.s. deviations = 0.01 Å]. In the crystal, Fig. 2, molecules are linked *via* intermolecular C–H···O hydrogen bonding interactions (Table 1) and π···π stacking interactions involving the benzene and maleinimide rings (Table 2), which is different form the similar compound (Shao, *et al.*, 2012).

### 2. Experimental

A suspension of *N*-(2-bromoethyl)phthalimide (2.54 g, 10.0 mmol), *N*-methylpiperazine (1.10 g, 11.1 mmol) and K_2_CO_3_ (2.70 g, 19.6 mmol) in 20 ml CH_3_CN was stirred at room temperature for 0.5 h, and then heated to reflux for over 20 h. After cooling and filtration, the filter residue was washed with CH_3_CN. And the filtrate and washing were combined prior to removing the solvent under vacuum. The title compound (2.15 g, 7.9 mmol) was obtained as colorless needles with m.p. 102.2–103.0 °C, after recrystallization from n-hexane. Colourless blocks were obtained by slow evaporation of CH_3_OH.

### 3. Refinement

All the H atoms were placed in geometrically idealized positions and constrained to ride on their parent atoms, with C–H distances of 0.93–0.97 Å, and with *U*_iso_(H) = 1.2*U*_eq_(C).

### Crystal data

**Table:**

C_15_H_19_N_3_O_2_	*Z* = 2
*M**_r_* = 273.33	*F*(000) = 292
Triclinic, *P*1	*D*_x_ = 1.254 Mg m^−^^3^
Hall symbol: -P 1	Mo *K*α radiation, λ = 0.71073 Å
*a* = 6.9537 (12) Å	Cell parameters from 2417 reflections
*b* = 8.4410 (15) Å	θ = 2.8–29.0°
*c* = 12.563 (2) Å	µ = 0.09 mm^−^^1^
α = 96.260 (4)°	*T* = 296 K
β = 98.381 (4)°	Block, colorless
γ = 92.647 (3)°	0.30 × 0.28 × 0.25 mm
*V* = 723.7 (2) Å^3^	

### Data collection

**Table:**

Bruker APEXII CCD diffractometer	2654 independent reflections
Radiation source: fine-focus sealed tube	2206 reflections with *I* > 2σ(*I*)
Graphite monochromator	*R*_int_ = 0.022
φ and ω scans	θ_max_ = 25.5°, θ_min_ = 1.7°
Absorption correction: multi-scan (*SADABS*; Bruker, 2000)	*h* = −8→8
*T*_min_ = 0.975, *T*_max_ = 0.979	*k* = −10→9
4194 measured reflections	*l* = −7→15

### Refinement

**Table:**

Refinement on *F*^2^	Secondary atom site location: difference Fourier map
Least-squares matrix: full	Hydrogen site location: inferred from neighbouring sites
*R*[*F*^2^ > 2σ(*F*^2^)] = 0.047	H-atom parameters constrained
*wR*(*F*^2^) = 0.154	*w* = 1/[σ^2^(*F*_o_^2^) + (0.0987*P*)^2^ + 0.097*P*] where *P* = (*F*_o_^2^ + 2*F*_c_^2^)/3
*S* = 1.01	(Δ/σ)_max_ < 0.001
2654 reflections	Δρ_max_ = 0.17 e Å^−^^3^
183 parameters	Δρ_min_ = −0.18 e Å^−^^3^
0 restraints	Extinction correction: *SHELXL97* (Sheldrick, 2008), Fc^*^=kFc[1+0.001xFc^2^λ^3^/sin(2θ)]^-1/4^
Primary atom site location: structure-invariant direct methods	Extinction coefficient: 0.074 (13)

### Special details

**Table:**

Geometry. All e.s.d.'s (except the e.s.d. in the dihedral angle between two l.s. planes) are estimated using the full covariance matrix. The cell e.s.d.'s are taken into account individually in the estimation of e.s.d.'s in distances, angles and torsion angles; correlations between e.s.d.'s in cell parameters are only used when they are defined by crystal symmetry. An approximate (isotropic) treatment of cell e.s.d.'s is used for estimating e.s.d.'s involving l.s. planes.
Refinement. Refinement of *F*^2^ against ALL reflections. The weighted *R*-factor *wR* and goodness of fit *S* are based on *F*^2^, conventional *R*-factors *R* are based on *F*, with *F* set to zero for negative *F*^2^. The threshold expression of *F*^2^ > σ(*F*^2^) is used only for calculating *R*-factors(gt) *etc*. and is not relevant to the choice of reflections for refinement. *R*-factors based on *F*^2^ are statistically about twice as large as those based on *F*, and *R*- factors based on ALL data will be even larger.

### Fractional atomic coordinates and isotropic or equivalent isotropic displacement parameters (Å^2^)

**Table:**

	*x*	*y*	*z*	*U*_iso_*/*U*_eq_	
C1	0.7472 (2)	0.1040 (2)	0.64462 (16)	0.0663 (5)	
H1	0.7495	0.1864	0.7004	0.080*	
C2	0.7384 (3)	−0.0535 (3)	0.6639 (2)	0.0853 (7)	
H2	0.7335	−0.0780	0.7339	0.102*	
C3	0.7366 (3)	−0.1743 (3)	0.5820 (3)	0.0952 (9)	
H3	0.7321	−0.2795	0.5976	0.114*	
C4	0.7415 (3)	−0.1437 (2)	0.4756 (2)	0.0866 (8)	
H4	0.7398	−0.2263	0.4200	0.104*	
C5	0.7489 (2)	0.0147 (2)	0.45574 (15)	0.0583 (5)	
C6	0.75250 (19)	0.13529 (18)	0.53922 (13)	0.0490 (4)	
C7	0.7637 (2)	0.28929 (18)	0.49368 (12)	0.0470 (4)	
C8	0.7547 (2)	0.0895 (2)	0.35403 (15)	0.0623 (5)	
C9	0.7949 (2)	0.3733 (3)	0.31185 (14)	0.0682 (5)	
H9A	0.8471	0.3229	0.2500	0.082*	
H9B	0.8920	0.4539	0.3501	0.082*	
C10	0.6130 (3)	0.4551 (2)	0.27092 (14)	0.0618 (5)	
H10A	0.5547	0.4975	0.3328	0.074*	
H10B	0.6512	0.5447	0.2348	0.074*	
C11	0.5329 (2)	0.3035 (2)	0.09326 (13)	0.0585 (4)	
H11A	0.6436	0.2385	0.1059	0.070*	
H11B	0.5750	0.3973	0.0624	0.070*	
C12	0.3737 (3)	0.2097 (2)	0.01439 (13)	0.0613 (5)	
H12A	0.4212	0.1801	−0.0534	0.074*	
H12B	0.3373	0.1124	0.0431	0.074*	
C13	0.1346 (2)	0.3462 (2)	0.09674 (13)	0.0534 (4)	
H13A	0.0968	0.2504	0.1267	0.064*	
H13B	0.0211	0.4083	0.0848	0.064*	
C14	0.2917 (2)	0.44238 (19)	0.17559 (12)	0.0532 (4)	
H14A	0.3254	0.5402	0.1467	0.064*	
H14B	0.2430	0.4712	0.2432	0.064*	
C15	0.0515 (3)	0.2147 (3)	−0.08443 (17)	0.0777 (6)	
H15A	0.0143	0.1167	−0.0585	0.117*	
H15B	0.0996	0.1915	−0.1519	0.117*	
H15C	−0.0595	0.2779	−0.0951	0.117*	
N1	0.46613 (18)	0.35309 (15)	0.19655 (9)	0.0484 (3)	
N2	0.20302 (19)	0.30264 (16)	−0.00548 (10)	0.0541 (4)	
N3	0.76325 (17)	0.25325 (17)	0.38367 (10)	0.0521 (4)	
O1	0.77364 (19)	0.42351 (14)	0.54040 (10)	0.0671 (4)	
O2	0.7541 (2)	0.0268 (2)	0.26330 (12)	0.0968 (6)	

### Atomic displacement parameters (Å^2^)

**Table:**

	*U*^11^	*U*^22^	*U*^33^	*U*^12^	*U*^13^	*U*^23^
C1	0.0513 (9)	0.0830 (13)	0.0639 (11)	0.0043 (8)	−0.0028 (8)	0.0210 (9)
C2	0.0537 (11)	0.0913 (16)	0.1136 (18)	0.0011 (10)	−0.0070 (11)	0.0526 (15)
C3	0.0492 (11)	0.0704 (14)	0.164 (3)	0.0016 (9)	−0.0154 (13)	0.0502 (17)
C4	0.0445 (9)	0.0536 (11)	0.149 (2)	0.0062 (7)	−0.0133 (11)	−0.0109 (12)
C5	0.0338 (7)	0.0555 (9)	0.0795 (12)	0.0077 (6)	−0.0046 (7)	−0.0040 (8)
C6	0.0329 (7)	0.0546 (9)	0.0564 (9)	0.0042 (6)	−0.0025 (6)	0.0048 (7)
C7	0.0382 (7)	0.0555 (9)	0.0436 (8)	0.0009 (6)	−0.0003 (6)	−0.0014 (6)
C8	0.0384 (8)	0.0796 (12)	0.0612 (10)	0.0113 (7)	0.0005 (7)	−0.0197 (8)
C9	0.0503 (9)	0.1015 (14)	0.0504 (9)	−0.0138 (9)	0.0030 (7)	0.0131 (9)
C10	0.0672 (10)	0.0666 (10)	0.0485 (9)	−0.0100 (8)	0.0004 (7)	0.0109 (7)
C11	0.0535 (9)	0.0802 (11)	0.0439 (8)	0.0120 (8)	0.0104 (7)	0.0087 (8)
C12	0.0636 (10)	0.0726 (11)	0.0466 (9)	0.0210 (8)	0.0065 (7)	−0.0027 (8)
C13	0.0515 (9)	0.0584 (9)	0.0511 (9)	0.0121 (7)	0.0080 (7)	0.0067 (7)
C14	0.0598 (9)	0.0564 (9)	0.0442 (8)	0.0118 (7)	0.0090 (7)	0.0040 (7)
C15	0.0771 (13)	0.0777 (13)	0.0673 (12)	0.0170 (10)	−0.0150 (10)	−0.0104 (10)
N1	0.0503 (7)	0.0565 (8)	0.0385 (7)	0.0033 (5)	0.0062 (5)	0.0066 (5)
N2	0.0588 (8)	0.0588 (8)	0.0423 (7)	0.0119 (6)	0.0009 (6)	0.0015 (6)
N3	0.0424 (7)	0.0677 (9)	0.0434 (7)	0.0035 (5)	0.0020 (5)	−0.0005 (6)
O1	0.0850 (9)	0.0542 (7)	0.0564 (7)	0.0002 (6)	0.0027 (6)	−0.0058 (5)
O2	0.0825 (10)	0.1228 (13)	0.0717 (9)	0.0219 (9)	0.0034 (7)	−0.0439 (9)

### Geometric parameters (Å, º)

**Table:**

C1—C2	1.377 (3)	C10—H10A	0.9700
C1—C6	1.384 (3)	C10—H10B	0.9700
C1—H1	0.9300	C11—N1	1.466 (2)
C2—C3	1.366 (4)	C11—C12	1.504 (2)
C2—H2	0.9300	C11—H11A	0.9700
C3—C4	1.394 (4)	C11—H11B	0.9700
C3—H3	0.9300	C12—N2	1.459 (2)
C4—C5	1.386 (3)	C12—H12A	0.9700
C4—H4	0.9300	C12—H12B	0.9700
C5—C6	1.376 (2)	C13—N2	1.450 (2)
C5—C8	1.490 (3)	C13—C14	1.503 (2)
C6—C7	1.480 (2)	C13—H13A	0.9700
C7—O1	1.2120 (19)	C13—H13B	0.9700
C7—N3	1.382 (2)	C14—N1	1.464 (2)
C8—O2	1.202 (2)	C14—H14A	0.9700
C8—N3	1.388 (2)	C14—H14B	0.9700
C9—N3	1.458 (2)	C15—N2	1.451 (2)
C9—C10	1.519 (3)	C15—H15A	0.9600
C9—H9A	0.9700	C15—H15B	0.9600
C9—H9B	0.9700	C15—H15C	0.9600
C10—N1	1.457 (2)		
			
C2—C1—C6	117.6 (2)	C12—C11—H11A	109.4
C2—C1—H1	121.2	N1—C11—H11B	109.4
C6—C1—H1	121.2	C12—C11—H11B	109.4
C3—C2—C1	121.1 (2)	H11A—C11—H11B	108.0
C3—C2—H2	119.4	N2—C12—C11	111.05 (14)
C1—C2—H2	119.4	N2—C12—H12A	109.4
C2—C3—C4	121.6 (2)	C11—C12—H12A	109.4
C2—C3—H3	119.2	N2—C12—H12B	109.4
C4—C3—H3	119.2	C11—C12—H12B	109.4
C5—C4—C3	117.5 (2)	H12A—C12—H12B	108.0
C5—C4—H4	121.3	N2—C13—C14	110.46 (13)
C3—C4—H4	121.3	N2—C13—H13A	109.6
C6—C5—C4	120.36 (19)	C14—C13—H13A	109.6
C6—C5—C8	107.93 (15)	N2—C13—H13B	109.6
C4—C5—C8	131.71 (19)	C14—C13—H13B	109.6
C5—C6—C1	121.86 (17)	H13A—C13—H13B	108.1
C5—C6—C7	107.87 (15)	N1—C14—C13	111.53 (13)
C1—C6—C7	130.27 (15)	N1—C14—H14A	109.3
O1—C7—N3	124.58 (15)	C13—C14—H14A	109.3
O1—C7—C6	128.68 (14)	N1—C14—H14B	109.3
N3—C7—C6	106.73 (13)	C13—C14—H14B	109.3
O2—C8—N3	124.7 (2)	H14A—C14—H14B	108.0
O2—C8—C5	129.28 (19)	N2—C15—H15A	109.5
N3—C8—C5	106.01 (14)	N2—C15—H15B	109.5
N3—C9—C10	114.32 (13)	H15A—C15—H15B	109.5
N3—C9—H9A	108.7	N2—C15—H15C	109.5
C10—C9—H9A	108.7	H15A—C15—H15C	109.5
N3—C9—H9B	108.7	H15B—C15—H15C	109.5
C10—C9—H9B	108.7	C10—N1—C14	108.35 (13)
H9A—C9—H9B	107.6	C10—N1—C11	112.08 (13)
N1—C10—C9	114.94 (15)	C14—N1—C11	108.84 (12)
N1—C10—H10A	108.5	C13—N2—C15	111.53 (14)
C9—C10—H10A	108.5	C13—N2—C12	108.61 (12)
N1—C10—H10B	108.5	C15—N2—C12	111.25 (13)
C9—C10—H10B	108.5	C7—N3—C8	111.45 (15)
H10A—C10—H10B	107.5	C7—N3—C9	123.36 (15)
N1—C11—C12	111.26 (13)	C8—N3—C9	124.69 (15)
N1—C11—H11A	109.4		

### Hydrogen-bond geometry (Å, º)

**Table:**

*D*—H···*A*	*D*—H	H···*A*	*D*···*A*	*D*—H···*A*
C3—H3···O1^i^	0.93	2.57	3.406 (3)	149
C15—H15*B*···O2^ii^	0.96	2.53	3.343 (3)	142

Symmetry codes: (i) *x*, *y*−1, *z*; (ii) −*x*+1, −*y*, −*z*.

### Table 2 π-π hydrogen-bond geometry (Å)

**Table:**

*Cg*I	*Cg*J	*Cg*I···*Cg*J
*Cg*1	*Cg*3^i^	3.607 (1)
*Cg*1	*Cg*3^ii^	3.716 (1)
*Cg*1	*Cg*3^i^	3.556 (1)

Symmetry codes: (i) 1-*x*, -*y*, 1-*z*; (ii) 2-*x*, -*y*, 1-*z*; *Cg*1 and *Cg*3 are the centroids of the N3–C7–C6–C5–C8 and C1–C6 rings.
